# Novel engineered, membrane-localized variants of vascular endothelial growth factor (VEGF) protect retinal ganglion cells: a proof-of-concept study

**DOI:** 10.1038/s41419-018-1049-0

**Published:** 2018-10-03

**Authors:** Junhui Shen, Ru Xiao, Jeffrey Bair, Fang Wang, Luk H. Vandenberghe, Darlene Dartt, Petr Baranov, Yin Shan Eric Ng

**Affiliations:** 1 Harvard Ophthalmology, Schepens Eye Research Institute of Massachusetts Eye and Ear, Boston, MA USA; 2 Department of Ophthalmology, Shanghai Tenth People’s Hospital, Tongji University School of Medicine, Shanghai, China; 3 Eye Center of the 2nd Affiliated Hospital, Zhejiang University School of Medicine, Hangzhou, China; 4 Grousbeck Gene Therapy Center, Ocular Genomics Institute, Mass Eye and Ear, Boston, MA USA; 5 The Broad Institute of Harvard and MIT, Cambridge, MA USA

## Abstract

Endogenous vascular endothelial growth factor (VEGF-A) can protect retinal ganglion cells (RGC) from stress-induced cell death in ocular hypertensive glaucoma. To exploit the neuroprotective function of VEGF-A for therapeutic application in ocular disorders such as glaucoma while minimizing unwanted vascular side effects, we engineered two novel VEGF variants, eVEGF-38 and eVEGF-53. These variants of the diffusible VEGF-A isoform VEGF121 are expressed as dimeric concatamers and remain tethered to the cell membrane, thus restricting the effects of the engineered VEGF to the cells expressing the protein. For comparison, we tested a Myc-tagged version of VEGF189, an isoform that binds tightly to the extracellular matrix and heparan sulfate proteoglycans at the cell surface, supporting only autocrine and localized juxtacrine signaling. In human retinal endothelial cells (hREC), expression of eVEGF-38, eVEGF-53, or VEGF189 increased VEGFR2 phosphorylation without increasing expression of pro-inflammatory markers, relative to VEGF165 protein and vector controls. AAV2-mediated transduction of eVEGF-38, eVEGF-53, or VEGF189 into primary mouse RGC promoted synaptogenesis and increased the average total length of neurites and axons per RGC by ~ 12-fold, an increase that was mediated by VEGFR2 and PI3K/AKT signaling. Expression of eVEGF-38 in primary RGC enhanced expression of genes associated with neuritogenesis, axon outgrowth, axon guidance, and cell survival. Transduction of primary RGC with any of the membrane-associated VEGF constructs increased survival both under normal culture conditions and in the presence of the cytotoxic chemicals H_2_O_2_ (via VEGFR2/PI3K/AKT signaling) and *N*-methyl-d-aspartate (via reduced Ca^2+^ influx). Moreover, RGC number was increased in mouse embryonic stem cell-derived retinal organoid cultures transduced with the eVEGF-53 construct. The novel, engineered VEGF variants eVEGF-38 and eVEGF-53 show promise as potential therapeutics for retinal RGC neuroprotection when delivered using a gene therapy approach.

## Introduction

Vascular endothelial growth factor (VEGF-A) is best known for its effects on vascular permeability and angiognenesis. Expression of receptors for VEGF-A was originally believed to be restricted to vascular endothelial cells. However, subsequent studies have strongly established a functional role for VEGF-A in neurons^1^. Most (if not all) neurons express neuropilins, receptors specific for the VEGF164 and VEGF188 isoforms^2^, in addition to VEGF receptor 2 (VEGFR2)^3,4^. In the eye, VEGF-A maintains homeostasis in retinal ganglion cells (RGC), which express VEGFR2^3,5^, and contributes to retinal disease states^6^. VEGF-A produced by RGC mediates protection from stress-induced cell death, including that from ocular hypertensive glaucoma and ischemia–reperfusion injury^5,7^. Both exogenous and endogenous VEGF-A have been shown to have potent neuroprotective effects on RGC in mouse models of neovascular age-related macular degeneration and diabetic retinopathy^8^. Interestingly, VEGF-A antagonism reduces axonal transport by RGC^8^, suggesting that VEGF-A can modulate function as well as survival of these retinal neurons^9^.

VEGF-A exists in several isoforms, including VEGF121, VEGF165, and VEGF189 in humans (corresponding to VEGF120, VEGF164, and VEGF188 in mice), and diffusible isoforms such as VEGF165 can induce unwanted blood vessel growth, vascular permeability, and inflammation in the retina^6,10,11^. To avoid these harmful effects, we developed two novel, engineered VEGF-A variants (eVEGF-38 and eVEGF-53) based on the VEGF121 isoform, which has been shown to be non-inflammatory^5,7,11^. These VEGF-A variants are designed to remain tethered to the plasma membrane, therefore acting only on the cells that express the proteins and preventing or limiting off-target effects of secreted and diffusible exogenous VEGF-A. We examined the effects of these engineered variants and those of the membrane-associated isoform VEGF189 on RGC morphology, function, and survival in vitro, using a gene delivery approach.

## Results

### The eVEGF variants, eVEGF-38 and eVEGF-53, are expressed by target cells as membrane-anchored dimers

**Fig. 1:**
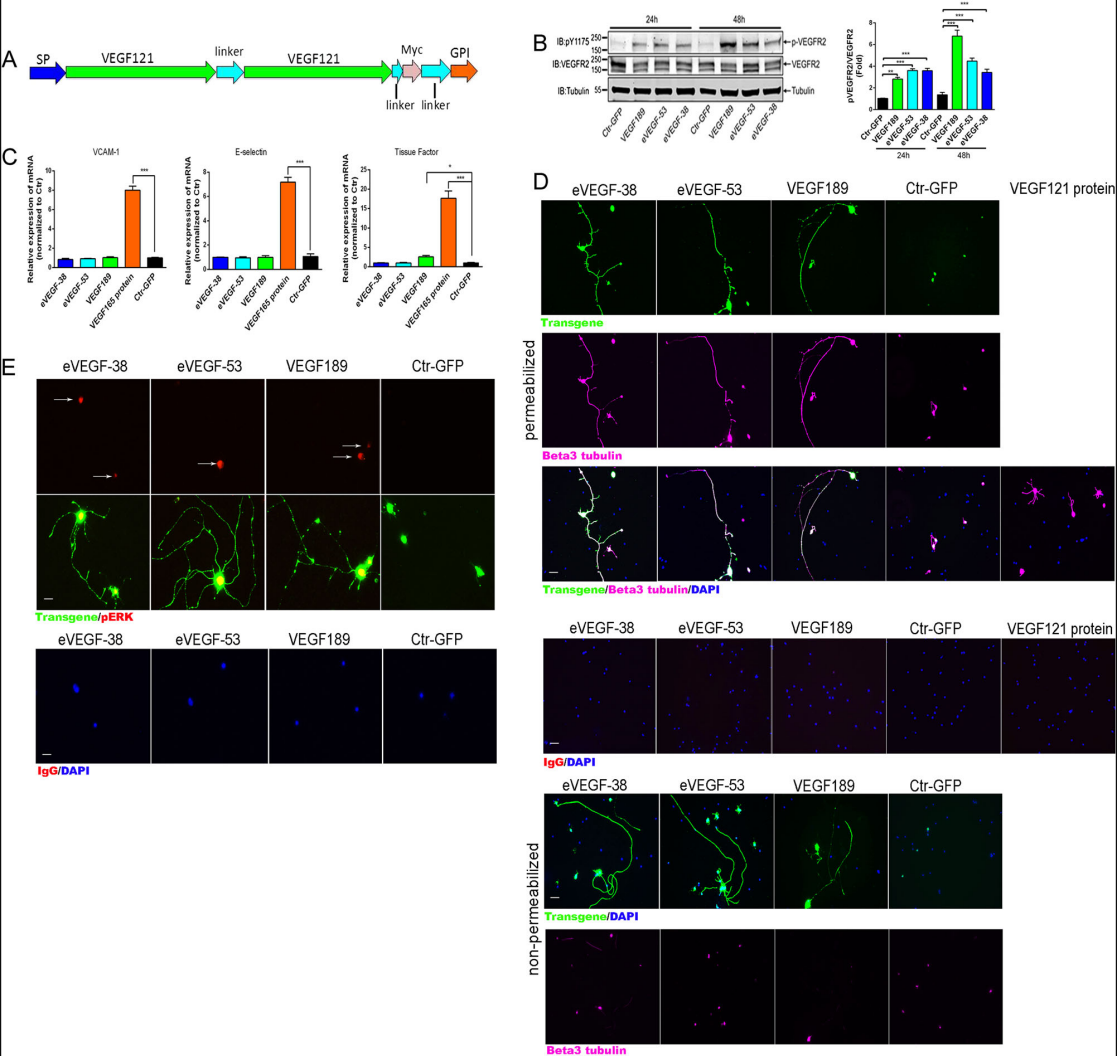
The eVEGF-38, eVEGF-53, and Myc-tagged VEGF189 proteins are localized to the cell surface and activate VEGFR2 signaling in both hREC and primary mouse RGC. **a** A schematic of an engineered VEGF construct (eVEGF), showing the location of the Myc epitope tag, the flexible linkers and signal for the GPI anchor. SP, signal peptide. **b** Sustained VEGFR2 activation following expression of eVEGF-38, eVEGF-53, and VEGF189 in hREC. The constructs and the GFP control were transfected into hREC. Left, western blot analysis with antibodies against phospho-VEGFR2 (pY1175), total VEGFR2, and alpha tubulin for total cell lysates obtained at 24 and 48 h post transfection. Right, VEGFR2 phosphorylation at 24 and 48 h post transfection. **P < 0.01, ***P < 0.001, unpaired two-tailed *t* test, *n* = three independent western blot experiments from three independent lysate samples, data = mean ± SEM. **c** Real-time qRT-PCR of pro-inflammatory genes expression in hREC 72 h after AAV transduction with eVEGF-38, eVEGF-53, VEGF189, or GFP control (ctr), or 2 h after treatment with VEGF165 protein (20 ng/ml). *P < 0.05, ***P < 0.001, unpaired two-tailed *t* test, *n* = three independent wells from three independent experiments, data = mean ± SEM. **d** Immunocytochemical localization of transgenic VEGF (anti-Myc, green) and βIII tubulin (far red, purple) (merge = white) in permeabilized (top rows) and non-permeabilized (bottom rows) primary mouse RGC (P3) 3 days after AAV2-mediated transduction of eVEGF-38, eVEGF-53, VEGF189, and GFP. The engineered VEGF proteins were clearly detected by immunostaining in both the permeabilized and non-permeabilized samples at a comparable level, whereas the intracellular beta III tubulin protein expression was only detected in the permeabilized samples. Longer neurites are seen on the RGC expressing the VEGF constructs, compared with RGC treated with recombinant VEGF121 protein (105 ng/ml for 24 h). Note that the GFP expression was detected by auto-fluorescence of the GFP protein. Scale bar = 20 µm. **e** Immunocytochemical localization of transgenic VEGF (anti-Myc, green) and phosphorylated ERK (pERK, arrows, red) in primary mouse RGC (P3) 3 days after AAV2-mediated transduction of eVEGF-38, eVEGF-53, VEGF189, and GFP. Scale bar = 10 µm

To restrict the effects of the eVEGF variants to the targeted RGC, a glycophosphatidylinositol (GPI) anchor was used to tether the protein to the plasma membrane, an established protein engineering approach for this objective^12^. To prevent heterodimerization with endogenous VEGF-A monomers, the VEGF121 segments were fused to form an anti-parallel dimer (a concatamer with head-to-tail orientation), connected by a flexible linker. A Myc epitope tag was inserted near the GPI linker to allow localization of the eVEGF protein. The general design of the engineered VEGF protein dimer is shown in Fig. 1a.

As the optimal distance for binding to cell surface receptors was not known, two different versions of eVEGF, eVEGF-38 and eVEGF-53, were created with flexible linkers 38 and 53 amino acids in length, respectively, between the GPI anchor and the C-terminal of the VEGF dimer. These lengths were based on the estimated length of the VEGF-A-binding domain of VEGFR2 relative to the cell surface^13^. To see if membrane tethering would yield different results from close extracellular localization of the naturally occurring VEGF-A, we created a Myc-tagged version of the VEGF189 isoform. VEGF189, once secreted, binds tightly to heparan sulfate proteoglycans at the cell surface and to extracellular matrix; this isoform remains largely localized to the cells that produce it^10,14^. Secreted VEGF189 can be released from heparan sulfate proteoglycans and the extracellular matrix to promote angiogenesis and affect the vasculature^15^, but it had not been shown if VEGF189 induces retinal inflammation. The cDNAs for eVEGF-38, eVEGF-53, and VEGF189 were subcloned into an adeno-associated virus (AAV)-based expression vector for transfection and transduction, and the corresponding vector containing complementary DNA (cDNA) for green fluorescent protein (GFP) was used as a negative control^16^.

To demonstrate expression, HEK-293T cells were transfected with the eVEGF-38, eVEGF-53, VEGF189, and GFP constructs, then the levels of the corresponding mRNA and proteins were determined by quantitative reverse transcriptase polyerase chain reaction (qRT-PCR) and western blot analysis, respectively. At 24 h post transfection, the VEGF mRNA was robustly expressed, and at 48 h the Myc tags for eVEGF-38, eVEGF-53, and VEGF189 were detected in total cell lysates (Supplemental Figure 1). The molecular weights of ~ 55 kDa for the recombinant eVEGF-38 and eVEGF-53 dimers and ~ 26 kDa for the recombinant VEGF189 monomer are consistent with the predicted molecular weights for each recombinant protein.

### Expression of eVEGF-38, eVEGF-53, and VEGF189 activates VEGFR2 in a cell-autonomous fashion

To determine whether the recombinant eVEGF-38, eVEGF-53, and VEGF189 proteins were functional, human retinal endothelial cells (hREC), which express high levels of VEGFR2, were transfected with each VEGF construct separately and the GFP control. VEGF mRNA was robustly expressed by the cells transfected with the VEGF constructs, and immunoprecipitation and western blot analysis confirmed the expression of eVEGF-38 and eVEGF-53 protein in the total cell lysates (Supplemental Fig. 2). Only a negligible amount (~ 5% of total) of each protein was detected in the conditioned medium, consistent with localization to the cell membrane or surface. Similarly, the majority of the myc-tagged VEGF189 protein was detected in the total cell lysate. Slightly more VEGF189 was detected in the conditioned media compared to the eVEGF proteins (Supplemental Fig. 2), an observation that is consistent with the fact that VEGF189 is secreted while the eVEGF proteins are tethered to the membrane. Phosphorylation of VEGFR2 was observed at both 24 and 48 h post transfection (Fig. 1b), suggesting cell-autonomous VEGFR2 activation and signaling in hREC.

To enhance the ability to deliver the VEGF constructs, the eVEGF-38, eVEGF-53, Myc-tagged VEGF189, and GFP constructs were packaged into adeno-associated virus 2 (AAV2)^17^. To confirm that expression of the VEGF constructs does not stimulate inflammation, transduced hREC were evaluated for expression of pro-inflammatory genes. For hREC transduced with the VEGF constructs, there was little or no change in expression of the genes encoding vascular cell adhesion molecule 1, E-selectin, and tissue factor (Fig. 1c). As a positive control, we assessed the effect of VEGF165 protein and found that the expression of all three pro-inflammatory genes was significantly increased (Fig. 1c).

We next investigated the effects of the VEGF constructs on primary mouse RGC in vitro. 4 h after plating, RGC were transduced with eVEGF-38, eVEGF-53, VEGF189, or GFP. Transgene expression was confirmed by qRT-PCR and immunocytochemistry for the Myc epitope tag, and beta III tubulin was used as the RGC marker to confirm purity of the primary culture (Fig. 1d, Supplemental Figs. 3 and 4). Transduction efficiency was ~ 80% for each construct. In non-permeabilized cells, strong staining for the Myc tag was detected at the cell surface for RGC transduced with VEGF constructs (Fig. 1d), indicating that the eVEGF proteins are membrane-bound and presented to the extracellular space.

To determine whether transduction with the VEGF constructs leads to activation of signaling downstream of VEGFR2, phospho-Erk expression was evaluated by immunocytochemistry. As shown in Fig. 1e, strong phospho-Erk staining in the cell bodies was observed in eVEGF-38-, eVEGF-53-, and VEGF189-expressing RGC compared with control RGC-expressing GFP. Furthermore, expression of VEGF constructs resulted in RGC with significantly longer neurites and axons compared with GFP-transduced or VEGF121 protein-treated RGC (Fig. 1d, e), indicating that expression of the membrane-associated VEGF constructs has a cell-autonomous effect on neuronal phenotype of the RGC.

### Expression of eVEGF-38, eVEGF-53, and VEGF189 increases RGC number in two- and three-dimensional cultures

**Fig. 2:**
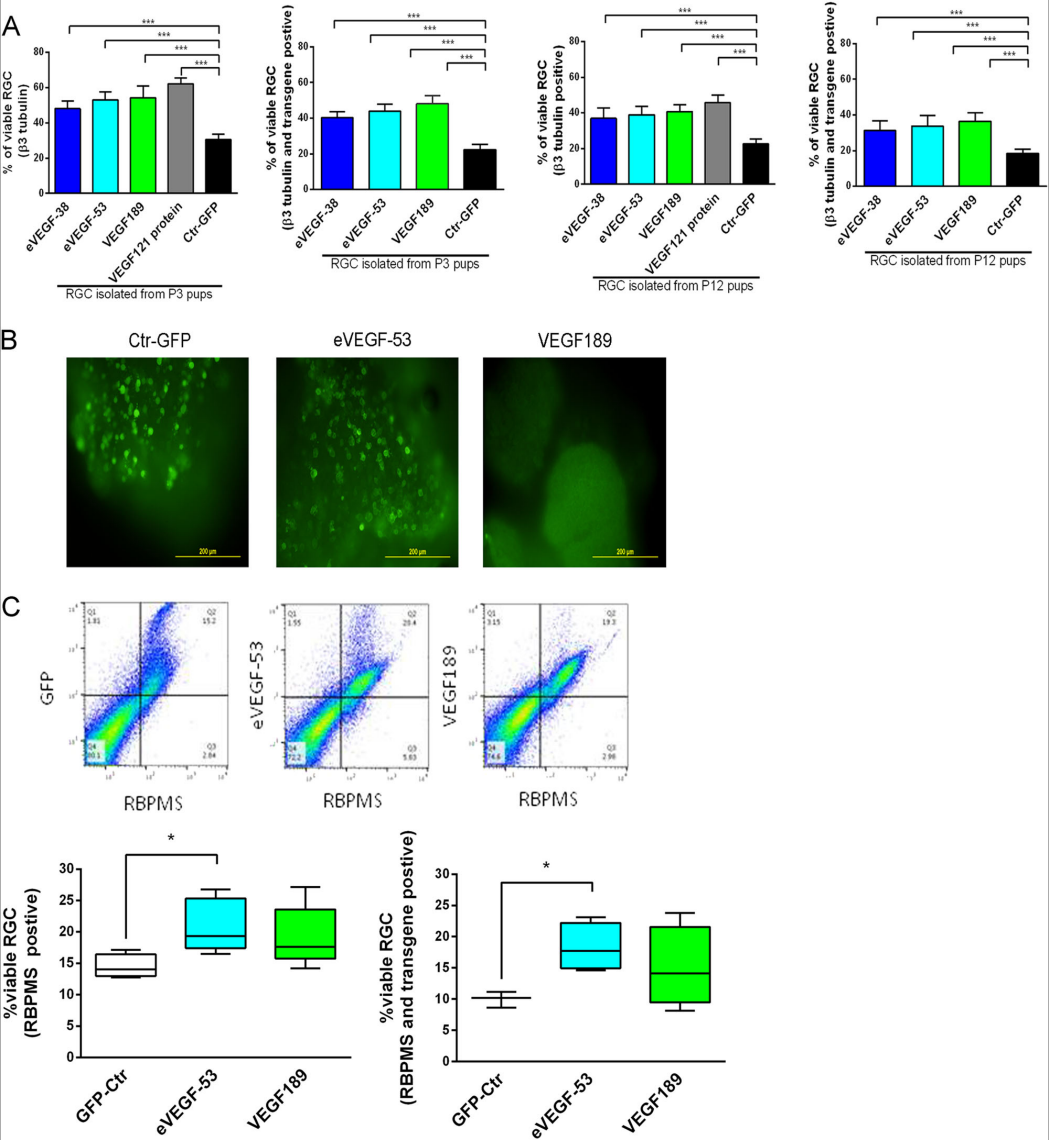
Expressed eVEGF-38, eVEGF-53, or VEGF189 increases RGC number in two-dimensional and three-dimensional cultures. **a** Viability of primary mouse RGC in culture, as measured by counting beta III tubulin-positive cells (viable RGC) and Myc + beta III tubulin double-positive cells (viable, transgene-expressing RGC), 3 days after transduction with the VEGF constructs or GFP. RGC were isolated from P3 and P12 mice. The primary RGC were treated with VEGF121 protein (105 ng/ml for 24 h) for comparison. ***P < 0.001, unpaired two-tailed *t* test, *n* = 15; five different fields from three independent experiments. **b** Representative immunostaining images showing expression of eVEGF-53 (anti-Myc), VEGF189 (anti-Myc), and GFP (anti-GFP) in mouse embryonic stem cell-derived, three-dimensional retinal organoids at 7 days after transduction. Note the discrete cellular staining for the expression of GFP and eVEGF-53 vs. the diffuse extracellular matrix staining for the VEGF189 protein. **c** Top, representative flow cytometry analysis showing the percentage of viable RGC (RBPMS-positive) that also express the transduced transgene (Myc-positive for eVEGF-53 and VEGF189, GFP-positive for the control). Bottom, quantification of the flow cytometry analysis for the 3D retinal organoids transduced with the eVEGF-53, VEGF189, or GFP constructs. Data = mean ± SEM, *P < 0.05, unpaired two-tailed *t* test, *n* ≥ 3 independent experiments

We next tested the neuroprotective effects of the eVEGF-38, eVEGF-53, and VEGF189 constructs on primary mouse RGC isolated at postnatal day (P) 3 and P12. Survival rates after 3 days in vitro (DIV 3^18^) were determined for RGC transduced with each of the three VEGF constructs or GFP by double staining for beta III tubulin^19^, a marker for viable RGC, and the Myc tag expressed by the transgenes. In addition, the number of beta III tubulin-positive cells (viable RGC) and double-stained cells (viable RGC that expressed the transgene) were compared with the total number of DAPI-stained cells. As a positive control, non-transduced RGC were treated for 24 h with recombinant VEGF121, which has neuroprotective function, starting at DIV 2. Enhanced survival was observed for VEGF-treated or VEGF-expressing primary RGC isolated from both P3 and P12 mice, compared to the GFP-expressing controls (Fig. 2a). The VEGF constructs promoted cell-autonomous RGC survival in culture, as most of the viable RGC (83–89%) also expressed the transgenes (Fig. 2a).

To examine the neuroprotective effects of the VEGF constructs on RGC in a more in vivo-like model system, we transduced mouse embryonic stem cell-derived three-dimensional retinal organoids with eVEGF-53 or VEGF189, and then evaluated RGC survival. As both eVEGF constructs are very similar in their ability to activate VEGFR2 signaling, the eVEGF-53 construct was selected for this experiment because it had a higher virus titer than that of the eVEGF-38 construct. A majority of the transduced cells, which were mostly RGC, observed 7 days after transduction were located close to the surface of the 3D organoids (Fig. 2b). The efficiency of AAV2-mediated transduction of day 21 retinal organoids was lower than that observed for primary mouse RGC. Flow cytometric analysis of the cells in the 28-day organoids revealed similar transduction efficiencies for the two VEGF constructs, at ~ 13% of total cells (data not shown). Transduction with eVEGF-53 significantly increased the percentage of RGC in the organoids compared with the GFP control (*P* < 0.05, Fig. 2c). Transduction with VEGF189 also improved the percentage of RGC yield, but the increase was not statistically significant (*P* = 0.11). Close to 90% of the viable RGC (beta tubulin-positive cells) in the eVEGF-53-transduced group expressed the engineered VEGF protein (Myc-positive cells in Fig. 2c), strongly suggesting that eVEGF-53 expression promotes RGC yield or survival in a cell-autonomous manner. Similarly, ~ 80% of the viable RGC in the VEGF189-transduced group expressed the recombinant protein (Fig. 2c).

### Expression of eVEGF-38, eVEGF-53, and VEGF189 supports neuritogenesis, axon growth, and synaptogenesis in RGC

**Fig. 3:**
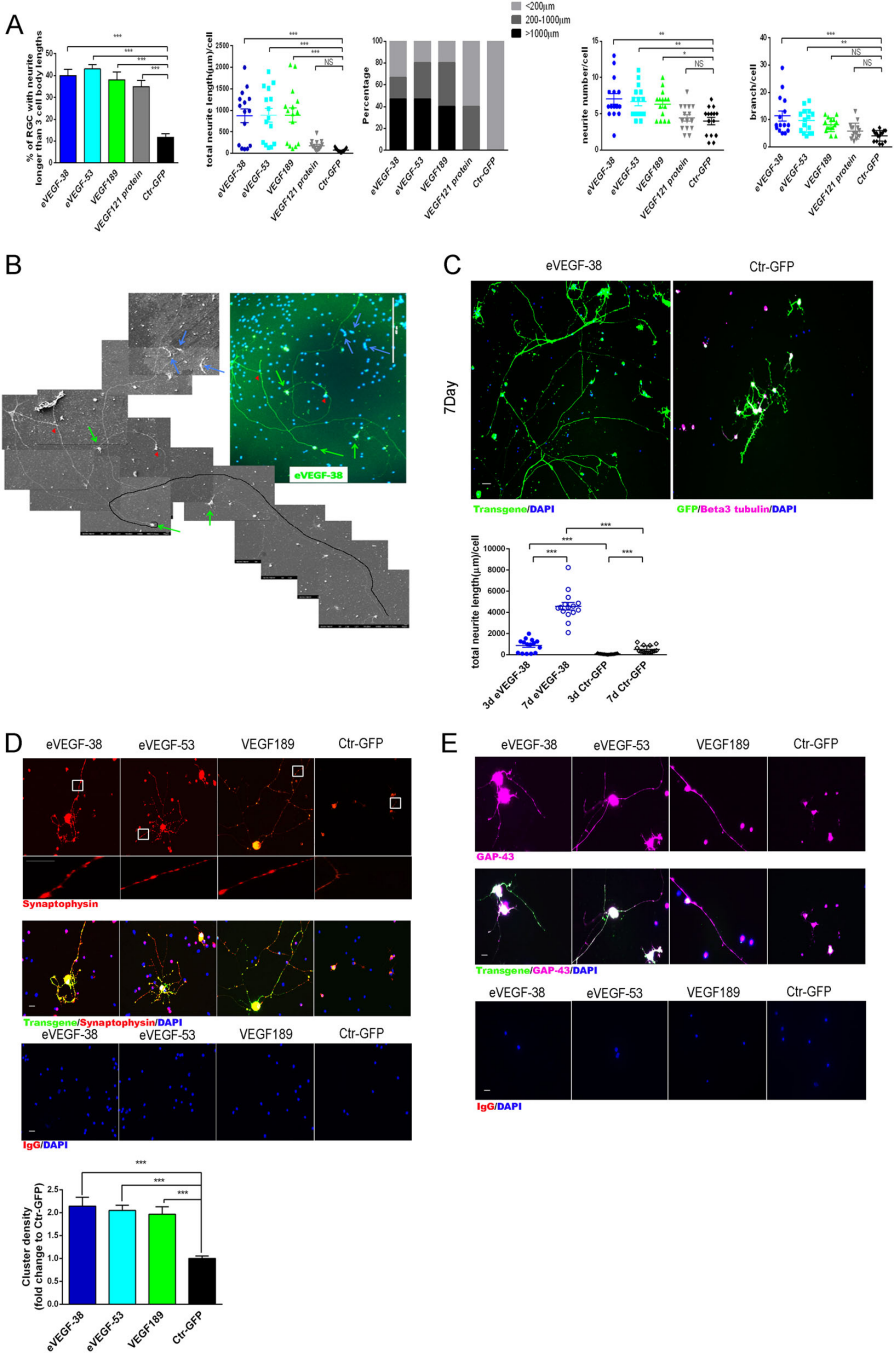
Expression of eVEGF-38, eVEGF-53, or VEGF189 supports neuritogenesis and axon outgrowth in primary mouse RGC. **a** Percentages of transduced RGC bearing neurites longer than three cell body lengths, average total neurite length per RGC, and distribution of neurite lengths per RGC, number of neurites per RGC and number of neurite branches per RGC were determined at days in vitro 3 (DIV 3). Neurite length was determined using image analysis software (Image J); neurite number and branch number were quantified using the same images. Immunostaining was used to identify RGC and neurites (anti-beta III tubulin), transgene expression (anti-Myc tag), and GFP expression (anti-GFP antibody). Primary RGC were treated with VEGF121 protein (105 ng/ml for 24 h) for comparison. *P < 0.05, **P < 0.01, ***P < 0.001, one-way ANOVA, *n* = 15; five different fields from three independent experiments. **b** A representative immunofluorescent correlative scanning EM image (× 400 magnification) showing eVEGF-38-expressing RGC (green arrows) with long axons (one axon outlined with black line). Possible synapses are indicated with the red arrowheads, and RGC not expressing the transgene are marked with blue arrows. **c** Outgrowth of neurites and axons in primary RGC transduced with eVEGF-38, at DIV 3 and DIV 7. Top, immunocytochemistry of the transduced primary RGC for eVEGF-38 (anti-Myc, green), βIII tubulin (purple), and GFP (green) on DIV 7. Scale bar = 50 µm. Bottom, quantification of average total neurite length per RGC at DIV 3 and DIV 7. ***P < 0.001, unpaired two-tailed *t* test, *n* = 15; five different fields from three independent experiments. **d** Immunocytochemical localization of synaptophysin (red) and eVEGF-38, eVEGF-53, or VEGF189 (anti-Myc, green, merged/colocalized = yellow). Note the increased punctate staining of synaptophysin on the cell bodies as well as the neurites and axons for RGC expressing the VEGF constructs, but not with the GFP control (white boxes, higher magnification inserts). Scale bar = 10 µm. The density of the synaptophysin-positive puncta per neurite increased significantly upon expression of the engineered VEGF constructs in primary RGC compared with the GFP control (graph). Data = mean ± SEM and normalized to GFP control, ***P < 0.001, unpaired two-tailed *t* test, *n* = 9 individual axon per group; three different RGC from three independent experiments. **e** Immunocytochemistry showing expression of GAP-43 (purple) by RGC-expressing eVEGF-38, eVEGF-53, or VEGF189 (anti-Myc, green; merged/colocalized = white). Note the strong GAP-43 staining colocalized with the recombinant VEGF on the cell bodies, neurites and axons. Scale bar = 10 µm. All RGC were isolated from P3 mice

To explore the effects of the VEGF constructs on RGC morphology, and expand on the observation that RGC expressing the eVEGF constructs had longer neurites and axons relative to the GFP control (Fig. 1), we tested the ability of eVEGF-38, eVEGF-53, and VEGF189 to induce outgrowth of neurites and axons. Immunocytochemical staining and morphometric analysis was used to quantify the length of the neurites and axons of transduced RGC on DIV 3. The percentages of RGC with at least one neurite longer than three times the length of the cell body, as an initial assessment of neurite outgrowth^20^, was significantly increased in cells expressing VEGF constructs compared with the GFP control (*P* < 0.001, Fig. 3a). The average total neurite length per cell was also significantly increased for RGC transduced with the VEGF constructs compared with GFP control (*P* < 0.001).

Stratification of total neurite length per cell demonstrated that transduction with the VEGF constructs resulted in more RGC with long neurites (total neurite length per cell > 1000 μm), VEGF121 protein treatment resulted in more RGC with neurites intermediate in length (total neurite length per cell between 200 and 1000 μm), and transduction with the GFP control resulted in RGC with short neurites (< 200 μm) (Fig. 3a). Compared with the GFP control, the average number of neurites per cell and average number of neurite branches per cell were both significantly increased by expression of eVEGF-38 and eVEGF-53 (*P* < 0.01 and *P* < 0.001, respectively), whereas VEGF189 expression significantly increased the number of neurites (*P* < 0.05) but not the number of branches per RGC.

Correlative scanning electron microscopy (SEM) was used to examine morphology of the long axons for RGC transduced with eVEGF-38. On DIV 3, long axons (many over 1500 μm in length) were clearly visible on multiple eVEGF-38-expressing RGC, whereas non-transduced neighboring RGC not expressing eVEGF-38 had short axons and neurites (Fig. 3b and Supplementary Figure 5). Correlative SEM also revealed that some of the long axons made contacts with other RGC (Fig. 3b), possibly indicating formation of synapses. Immunostaining on DIV 7 revealed axons significantly longer than those from DIV 3 for both the control GFP group and the eVEGF-38 group (*P* < 0.001, Fig. 3c).

To further explore the potential for synapse formation, the transduced RGC were immunostained for synaptophysin, a synapse marker, and GAP-43, a component of the axon and pre-synaptic terminal. RGC transduced with eVEGF-38, eVEGF-53, or VEGF189 displayed an increased density of clusters positive for synaptophysin compared to the GFP control, with synaptophysin frequently colocalizing with the recombinant VEGF proteins on the neurites and axons of the RGC (Fig. 3d). Expression of GAP-43 was also readily detected in the neurites of the RGC transduced with the VEGF constructs (Fig. 3e), consistent with increased synaptogenic potential in the presence of the cell surface-associated VEGF proteins.

### Neurites and axons outgrowth induced by the eVEGF-38, eVEGF-53, and VEGF189 is mediated by the VEGFR2/PI3K/Akt and is associated with upregulation of genes and proteins known to contribute to neuritogenesis

**Fig. 4:**
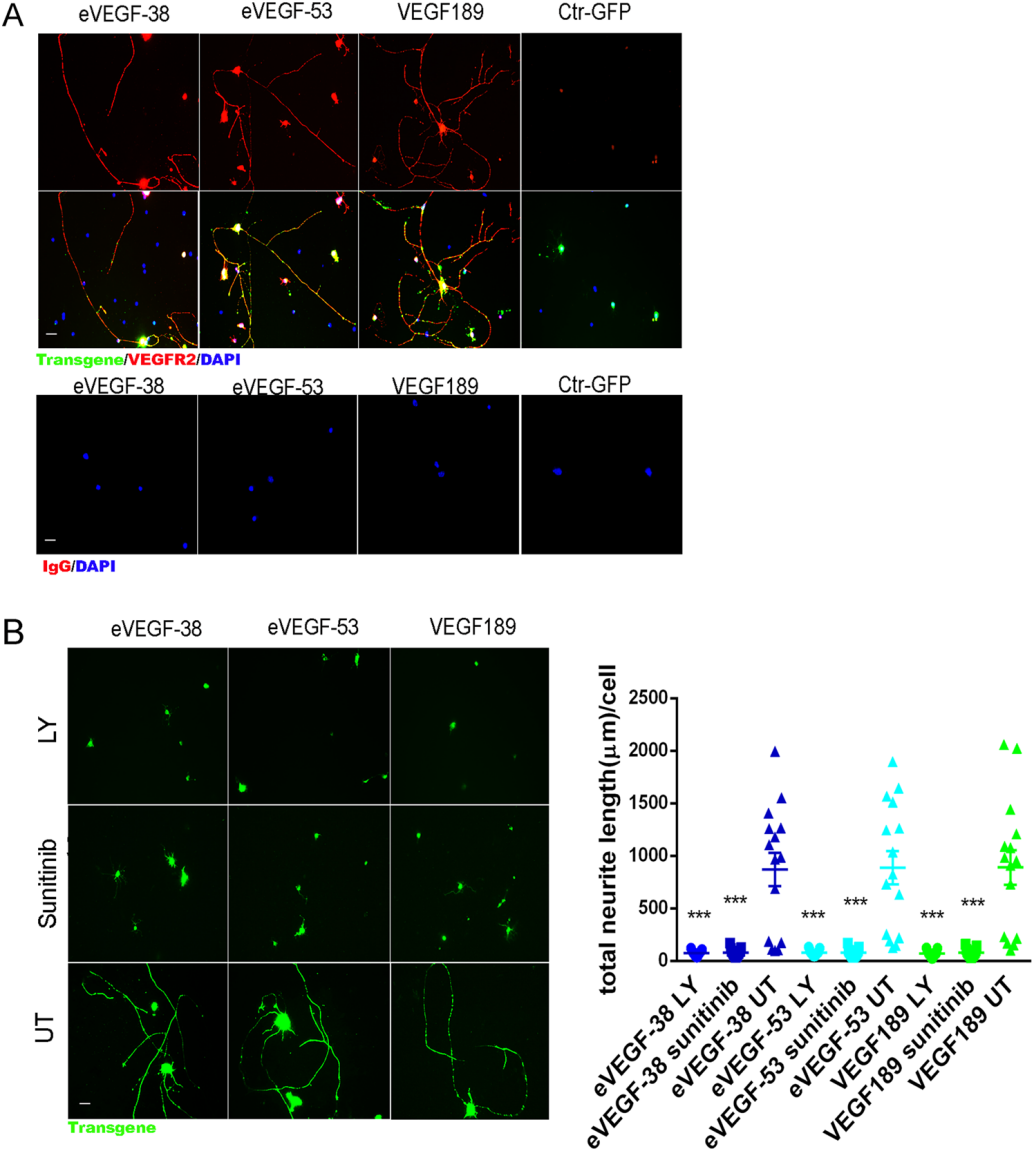
Outgrowth of neurites and axons induced by expression of eVEGF-38, eVEGF-53, or VEGF189 in primary mouse RGC is mediated by VEGFR2/PI3K/Akt pathway. **a** Representative immunocytochemistry showing VEGFR2 (red) expression and colocalization with eVEGF-38, eVEGF-53, VEGF189 (anti-Myc tag, green), or GFP (anti-GFP, green) on the cell bodies, neurites and axons of primary RGC at DIV 3. Note the lower levels of VEGFR2 expression and the lack of long neurites and axons in the RGC-expressing GFP. Scale bar = 20 µm. **b** Left, representative immunocytochemical localization of eVEGF-38, eVEGF-53, and VEGF189 (anti-Myc tag, green) outlining neurites and axons of RGC, in the presence or absence of the VEGFR2 inhibitor Sunitinib malate (1 μM) or the PI3K/AKT inhibitor LY294002 (LY, 10 μM), 3 days after AAV transduction. Scale bar = 20 µm, UT = untreated. Right, quantification of total neurite length per RGC in the presence or absence of Sunitnib malate or LY294002. ***P < 0.001 compared with the corresponding untreated (UT) control, unpaired two-tailed *t* test, *n* = 15; five different fields from three independent experiments. Data = mean ± SEM. All RGC were isolated from P3 mice

To examine the molecular mechanism(s) underlying the neuritogenesis observed with expression of VEGF constructs, we first focused on VEGFR2 signaling. Immunocytochemical staining showed that primary RGC transduced with eVEGF-38, eVEGF-53, or VEGF189 expressed high levels of VEGFR2, with strong staining detected on the cell bodies, neurites, and axons (Fig. 4a). Inhibition of VEGFR2 activation using the small-molecule inhibitor Sunitinib or inhibition of PI3K/AKT signaling using LY294002 completely inhibited neuritogenesis in RGC transduced with the VEGF constructs (*P* < 0.001 for treated vs. untreated, Fig. 4b).

**Fig. 5:**
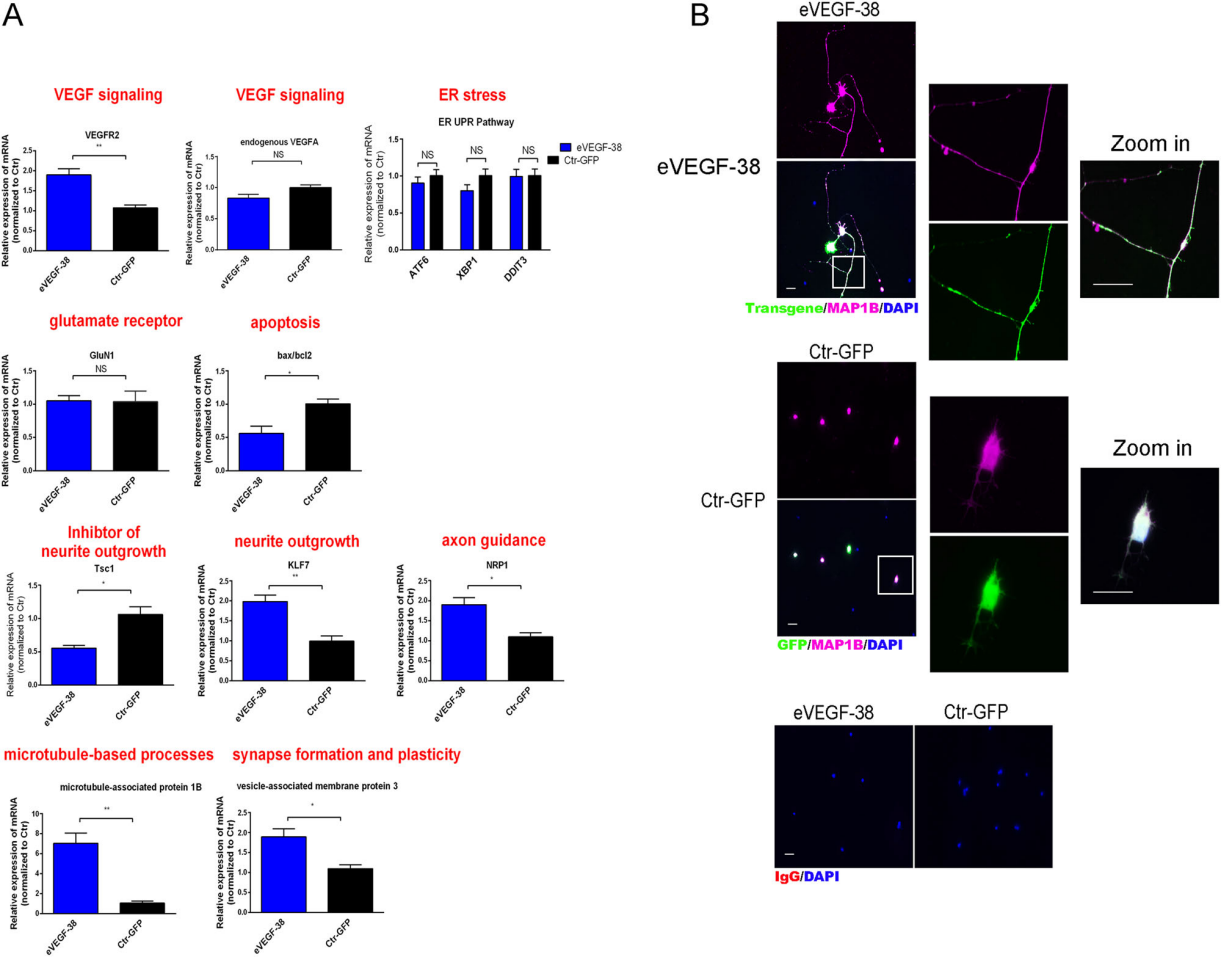
Expression of eVEGF-38 in primary mouse RGC induces genes that are involved in neurogenesis. **a** Quantification of gene expression by qRT-PCR in P4 RGC expressing the eVEGF-38 or GFP construct 3 days after AAV transduction. The expression levels for the genes encoding VEGFR2, endogenous VEGF-A, GluN1 NMDA receptor, Tsc1, KLF7, NRP-1, MAP1B, VAMP3, Bax, Bcl2, ATF6, XBP1, and DDIT3 were examined. Protein abbreviations are defined in the text. *P < 0.05, **P < 0.01, unpaired two-tailed *t* test, *n* = three independent experiments. Data = means ± SEM. **b** Immunocytochemistry of P4 primary mouse RGC transduced with the eVEGF-38 or GFP constructs for expression of MAP1B (purple), eVEGF-38 (anti-Myc tag, green), and GFP (anti-GFP, green). Note that both the long axons from the eVEGF-38-expressing RGC and the short neurites from the GFP-expressing RGC are positive for MAP1B staining. Left panels, scale bar = 20 µm, right panels, scale bar = 200 µm

We next examined the effects of membrane-associated VEGF on the expression of genes associated with neuritogenesis and neuronal function by comparing gene expression of eVEGF-38-transduced cells to that of GFP-transduced cells, as shown in Fig. 5a. Relative to the GFP-transduced controls, expression of eVEGF-38 induced a significant increase (*P* < 0.01) in the expression of the *VEGFR2* mRNA, but did not affect expression of endogenous *VEGF-A* or the gene for the *N*-methyl-d-aspartate (NMDA) receptor (*GluN1*). Expression of the gene for Tuberous sclerosis 1 (*TSC1*), which is associated with suppression of axon growth, was significantly reduced (0.56-fold, *P* < 0.05) in RGC transduced with eVEGF-38, whereas expression of the gene for Krüppel-like transcription factor 7 (*KLF7*), a promoter of axon growth, was significantly increased (*P* < 0.01), as was that of the gene for neuropilin-1 (*NRP-1*), a receptor involved in axon guidance (*P* < 0.05). The genes for microtubule-associated protein 1B (*MAP1B*), which is involved in microtubule assembly during neurogenesis, and vesicle-associated membrane protein 3 (*VAMP3*), which is involved in the fusion of synaptic vesicles, were expressed at significantly higher levels in RGC transduced with eVEGF-38 (*P* < 0.001 and *P* < 0.05, respectively), and the expression ratio of the genes for *BAX* and *BCL2*, which positively correlates with apoptosis activity, was significantly decreased (*P* < 0.05) in the RGC transduced with eVEGF-38. Expression of eVEGF-38 did not affect expression of genes involved in the unfolded protein response and endoplasmic reticulum (ER) stress, namely *ATF6* (the ATF6 pathway), *XBP1* (the IRE1 pathway), and *DDIT3* (the PERK pathway) (Fig. 5a)^21^.

To confirm the expression of one of the key genes for neuritogenesis at the protein level, immunocytochemical staining was used to examine localization of MAP1B. As shown in Fig. 5b, MAP1B colocalized with eVEGF-38 on the long axon of RGC transduced with the VEGF construct, whereas most MAP1B localized to the cell bodies and short axons of RGC transduced with GFP.

### Expression of eVEGF-38, eVEGF-53, and VEGF189 is neuroprotective for RGC

**Fig. 6:**
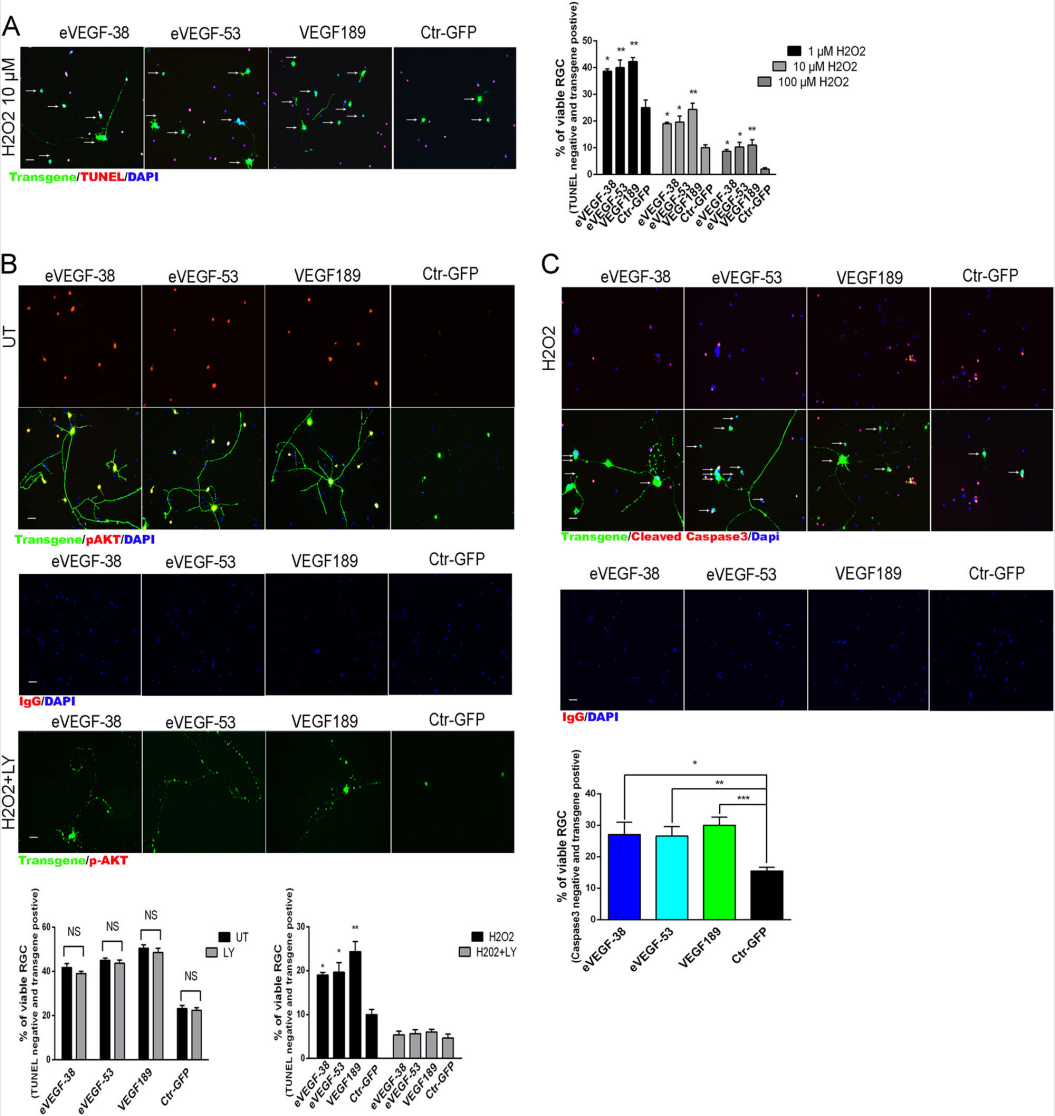
Expression of eVEGF-38, eVEGF-53, or VEGF189 in primary mouse RGC reduces cell death following exposure to H_2_O_2,_ a protective response that is VEGFR2/PI3K/AKT pathway-dependent. **a** TUNEL staining of primary mouse RGC transduced with the eVEGF-38, eVEGF-53, VEGF189, and GFP constructs. Transduced cells were treated with 1 µM, 10 µM, or 100 µM H_2_O_2_ for 5 h at DIV 3, then TUNEL stained (red) prior to staining for transgene expression (anti-Myc tag or anti-GFP, green). Left, representative images of RGC staining after H_2_O_2_ treatment (10 µM for 5 h). Viable RGC (TUNEL negative, transgene positive) are marked with arrows. Scale bar = 20 µm. Right, proportion of transduced, treated RGC that were viable and expressed the transgene (TUNEL-negative, transgene-positive) *P < 0.05, **P < 0.01, unpaired two-tailed *t* test compared with the corresponding GFP control, *n* = 15; five different fields from three independent experiments. **b** Activation of and requirement for P13K/AKT signaling in the protective response. Images, immunocytochemistry for transgene expression (anti-Myc tag or GFP, green) and phosphorylated AKT (red) in transduced RGC 3 days after AAV transduction with the eVEGF-38, eVEGF-53, VEGF189, and GFP constructs, in the presence or absence of H_2_O_2_ (10 µM) and the PI3K inhibitor LY294002 (LY, 10 µM). DAPI staining (blue) is used to visualize nuclei. Note the signs of degeneration visible in the RGC treated with LY294002 and H_2_O_2_. UT, untreated with LY294002 or H_2_O_2_. Scale bar = 20 µm. Graphs, proportions of transduced RGC that were viable and expressed the transgene (TUNEL-negative, transgene positive) with and without LY294002 (LY, 10 µM) and in the absence (left) or presence (right) of H_2_O_2_ (10 µM). *P < 0.05, **P < 0.01, unpaired two-tailed *t* test compared with the corresponding GFP control, *n* = 15; five different fields from three independent experiments. NS = not significant. **c** Reduction of cleaved caspase-3-positive primary mouse RGC transduced with the eVEGF-38, eVEGF-53, VEGF189 compared with GFP constructs in the presence of H_2_O_2_ (10 µM). Top panels, immunocytochemistry for activated, cleaved caspase-3 (red), and transgene expression (anti-Myc tag or anti-GFP, green). DAPI staining (blue) is used to visualize nuclei. Viable RGC (negative for cleaved caspase-3) are marked with arrows. Scale bar = 20 µm. The percentages of viable RGC (cleaved caspase-3-negative and transgene-positive and DAPI-positive) were significantly increased in RGC transduced with the eVEGF-38, eVEGF-53, and VEGF189 constructs compared with GFP controls in the presence of 10 µM H_2_O_2_. Data = mean ± SEM, *P < 0.05, **P < 0.01, ***P < 0.001, unpaired two-tailed *t* test compared with corresponding GFP, *n* = 9 per group; three different fields from three independent experiments. All RGC were isolated from P3 mice

As retinal degenerative diseases such as glaucoma involve death of RGC in response to stress, we examined the effects of the eVEGF-38, eVEGF-53, and VEGF189 constructs on RGC exposed to cytotoxic stimuli. To simulate oxidative stress, transduced mouse RGC were treated for 5 h with 1–100 µM H_2_O_2_, then cell death was quantified by transferase dUTP nick end labeling (TUNEL) staining^5^. Expression of eVEGF-38, eVEGF-53, or VEGF189 resulted in significantly higher RGC survival compared with the GFP control for all three H_2_O_2_ concentrations (*P* < 0.05, Fig. 6a).

We next explored the mechanism of the VEGF-mediated neuroprotection. As the PI3K/AKT pathway has previously been implicated in VEGFR2-mediated neuroprotection^5,22^, we examined activation of AKT in the transduced RGC by immunostaining. The number of phospho-AKT-positive cells was markedly increased in the RGC-expressing eVEGF-38, eVEGF-53, or VEGF189 compared with those expressing GFP (Fig. 6b). When the transduced RGC were treated with both H_2_O_2_ to induce cell death and LY294002 to suppress PI3K/AKT activation, phospho-AKT levels were greatly reduced and the RGC in all groups showed clear signs of increased cell death. Quantification of TUNEL-stained cells confirmed that PI3K/AKT inhibition abolished the protective effects of eVEGF-38, eEVGF-53, and VEGF189 on RGC exposed to H_2_O_2_. PI3K/AKT inhibition did not reduce the neuroprotection induced by the VEGF constructs under normal culture conditions (Fig. 6b), suggesting that the mechanism for cell survival/neuroprotection differs depending on the conditions. To determine the effect of the VEGF constructs expression on H_2_O_2_-induced caspase activation, which is involved in apoptosis of RGC in glaucoma^23^, immunocytochemistry was used to visualize cleaved (activated) caspase-3 expression in the H_2_O_2_-treated RGC. Indeed, fewer of the RGC-expressing VEGF constructs displayed cleaved caspase-3 compared with RGC-expressing GFP, and the percentage of viable RGC significantly increased in the RGC expressing the VEGF constructs compared with the GFP control (Fig. 6c).

**Fig. 7:**
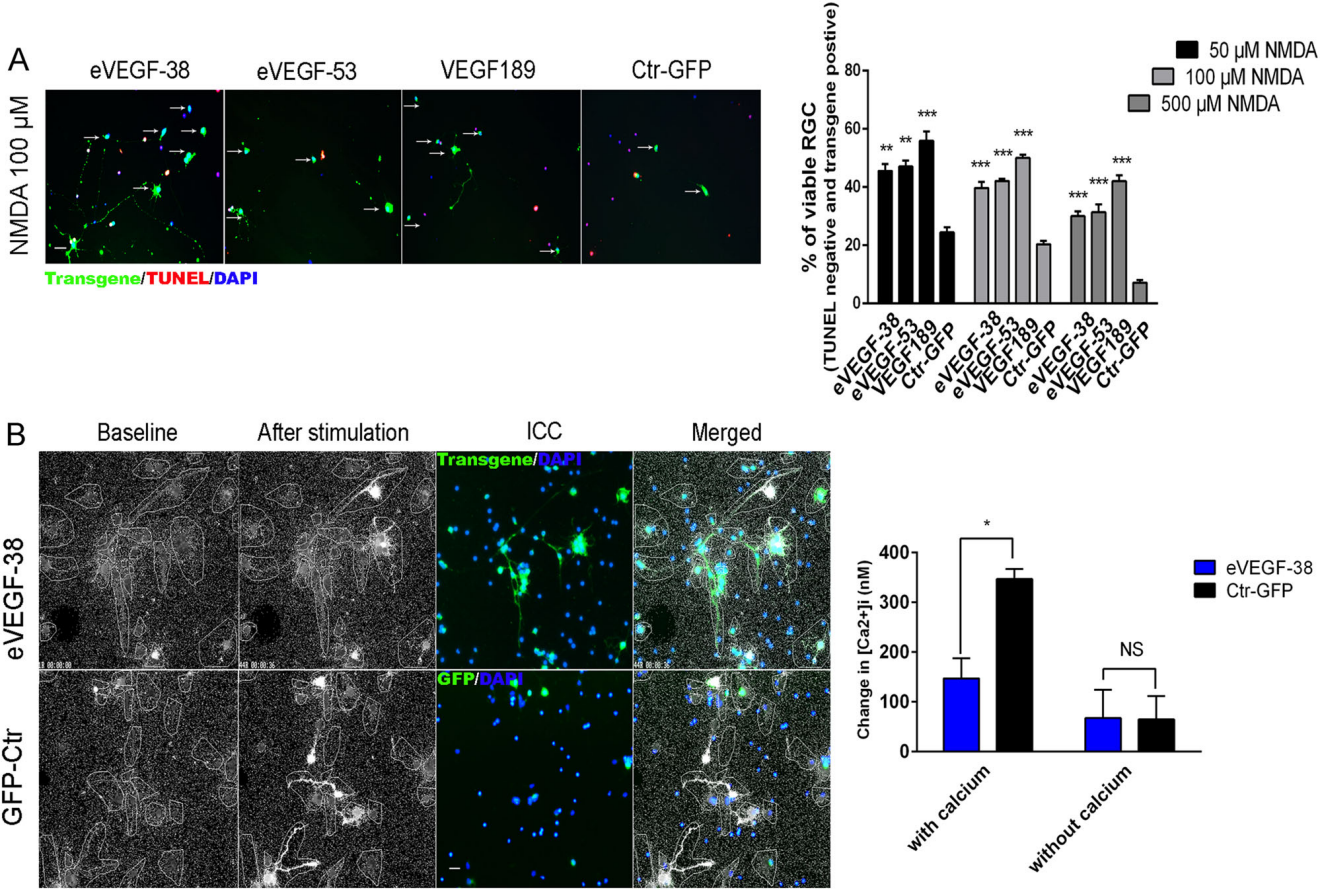
Expression of eVEGF-38, eVEGF-53, or VEGF189 in primary mouse RGC reduces cell death following exposure to the excitotoxic agent NMDA,_,_ a protective response that is associated with reduced calcium influx. **a** Survival of primary mouse RGC transduced with the eVEGF-38, eVEGF-53, VEGF189, and GFP constructs and treated the excitotoxic chemical NMDA. Left, representative immunocytochemistry images showing TUNEL staining (red) and expression of the transgenes (anti-Myc tag or anti-GFP, green) for transduced RGC treated with NMDA (100 µM) for 12 h. Viable RGC (TUNEL-negative and transgene-positive) are marked with arrows. Scale bar = 20 µm. All data = mean ± SEM. Right, transduced primary RGC were treated with 50, 100, or 500 µM NMDA for 12 h at DIV 2.5. TUNEL staining was used to assess viability in transgene-expressing RGC. **P < 0.01, ***P < 0.001, unpaired two-tailed *t* test compared with corresponding GFP, *n* = 15; five different fields from three independent experiments. **b** NMDA-induced calcium signaling in RGC-expressing eVEGF-38 or GFP. Left, representative live imaging of calcium signaling upon NMDA stimulation. Immunostaining for eVEGF-38 (anti-Myc tag, green) or GFP (anti-GFP, green) was conducted in the same RGC samples subsequent to assessment of calcium influx (white). Note in the merged panels that some of the eVEGF-38-expressing RGC display reduced calcium signaling. Right, quantification of calcium signaling (calcium influx, [Ca^2+^]i) upon NMDA stimulation in the presence or absence of 1 mM Ca^2+^ in the medium. *P < 0.05, unpaired two-tailed *t* test, *n* = three to four independent wells of primary RGC, Data = mean ± SEM. All RGC were isolated from P3 mice

NMDA type glutamate receptors contribute to synaptogenesis and synaptic plasticity, and when overactivated these receptors can induce excessive levels of Ca^2+^ influx and excitotoxicity. To examine the neuroprotective effects of the VEGF constructs in the context of excitotoxicity, transduced RGC were treated with NMDA for 12 h, and survival was evaluated using TUNEL staining. Expression of eVEGF-38, eVEGF-53, or VEGF189 resulted in significant protective effects against NMDA treatment at all doses tested, compared with the corresponding GFP control (*P* < 0.01 to *P* < 0.001, Fig. 7a; compare also with Fig. 2a with no treatment), indicating that the VEGF constructs are neuroprotective in the context of various cytotoxic stimuli.

We next determined whether the VEGF constructs reduced NMDA-induced cytotoxicity by decreasing Ca^2+^ influx. RGC transduced with eVEGF-38 displayed significantly reduced Ca^2+^ influx, as viewed by live imaging, when treated with NMDA compared the RGC transduced with GFP (*P* < 0.05, Fig. 7b); Ca^2+^ influx was not impacted by eVEGF-38 in the absence of extracellular calcium (*P* > 0.05). Immunostaining for eVEGF-38 in the RGC subsequent to live imaging revealed that most of the Ca^2+^ influx in the eVEGF-38-transduced cells occurred in cells that did not express the VEGF transgene (Fig. 7b, left).

## Discussion

The eVEGF-38, eVEGF-53, and myc-tagged VEGF189 constructs induced persistent activation of VEGFR2 and its downstream signaling pathways, an effect distinct from the transient VEGFR2 activation induced by soluble VEGF-A protein^24^. VEGFR2 was robustly expressed on the cell membrane of the transduced RGC, but we did not detect any substantial increase in the levels of intracellular or vesicle-associated VEGFR2. This might indicate reduction in internalization of the membrane-tethered eVEGF/VEGFR2 complex, which could contribute to the extended duration of signaling. It remains to be determined whether prolonged expression of the VEGF-A constructs and persistent activation of VEGFR2 beyond 7 days will result in suppression of VEGFR2 signaling; as occurs with neurons expressing brain-derived neurotrophic factor^25,26^.

The effects of the VEGF-A constructs on outgrowth of long neurites and axons (≥ 1500 μm per neurite) were unexpected; treatment with soluble VEGF121 in our model did not have a similar effect. Treatment with soluble VEGF165 has induced outgrowth of neurites in the superior cervical ganglia and dorsal root ganglia of mice that were limited in length (~ 500 μm)^27^. VEGFR2 might bind to the membrane-tethered eVEGF or membrane-associated VEGF189 and become “locked” into an active signaling complex on the RGC. Our observations that eVEGF-38, eVEGF-53, and VEGF189 colocalize with VEGFR2 at the cell membrane and that the VEGFR2 inhibitor Sunitinib completely inhibits neurite outgrowth are consistent with this hypothesis. The extended neurite outgrowth might also stem from enhanced survival, however.

The cell-autonomous protective activity illustrated in our assays has exciting potential, as does the lack of inflammatory gene expression by the cells expressing the VEGF constructs. The lack of inflammatory activity induced by VEGF121 has been previously documented^5,7,11^, but this is a novel finding for VEGF189. It remains to be determined how the VEGFR2 signaling pathway(s) induced by these constructs differ from those induced by soluble VEGF165 protein, which does stimulate expression of inflammatory genes.

Genes associated with cell survival, neurogenesis, synapse formation, and neuronal function were expressed at significantly higher levels in RGC expressing the eVEGF-38, findings that are consistent with reported functions for VEGF-A in neurons^27–30^. For example, eVEGF-38 expression significantly increased the mRNA levels of KLF7, an important transcription factor that can promote axon regeneration in postmitotic RGC; has been identified as a potential therapeutic target for promoting RGC axon regeneration in patients with glaucoma^31–33^. Expression of eVEGF-38 can clearly modulate genes and pathways that are important for RGC functions in addition to cell survival, and it is likely that eVEGF-53 will have similar effects.

The VEGF constructs did not induce any detectable ER stress or unfolded protein response in primary RGC, and the survival effects were maintained over 7 days. Survival effects were observed in the 3D retinal organoid culture model despite the lower transduction efficiencies, and in response to multiple cytotoxic stimuli. Interestingly, the suppression of NMDA-induced cell death and calcium influx in RGC-expressing eVEGF-38 did not involve reduced expression of the NMDA receptor GluN1. The eVEGF constructs might be of benefit in suppressing excitotoxic effects in diseases such as glaucoma and retinal ischemia.

Our findings show clear beneficial effects of eVEGF-38, eVEGF-53, and Myc-tagged VEGF189 in supporting survival and axonal outgrowth of RGC. The constructs were delivered effectively using AAV2 transduction and acted in a cell-autonomous fashion. Evaluation of protective effects of eVEGF-38 and eVEGF-53 in animal models of glaucoma or other diseases involving RGC degeneration is clearly warranted.

## Methods

### HEK293 T cells and hREC culture

Human embryonic kidney (HEK) 293 T cells (ThermoFisher Scientific, Rockford, IL) were cultured in Dulbecco's Modified Eagle's Medium (Gibco; Invitrogen, Carlsbad, CA) supplemented with 10% fetal bovine serum (FBS). Primary human retinal microvascular endothelial cells (hREC) were purchased from Cell Systems (Kirkland, WA) and cultured in an endothelial growth medium (EGM)-2 kit (Lonza, Walkersvile, MD) and 2% FBS (Atlanta Biologicals, Flowery Branch, GA). All cells were maintained at 37 °C in a humidified incubator with 5% CO_2_.

### DNA constructs

All three cDNA constructs (eVEGF-38, eVEF-53, VEGF189) were synthesized (gBlock custom DNA synthesis, Integrated DNA Technologies, San Diego, CA) and subcloned into the GFP-containing pAAV2/2.CMV.EGFP.WPRE.BGH vector^16^ with a modified CMV promoter. The NotI and BamHI restriction sites were used to replace the cDNA for the EGFP with that of the VEGF constructs. All three constructs were verified by DNA sequencing. The parental vector was used as the GFP control.

### Transient transfection of HEK-293T and hREC

HEK-293T cells were transfected at 80% confluence using the TransIT-293 Transfection Reagent (Mirus Bio, Madison, WI) according to the manufacturer’s instructions (2.5 µg plasmid DNA and 7.5 µL TransIT-293 reagent per well). At 24 h and 48 h post transfection, HEK-293T cells were collected for extraction of RNA and protein.

The hREC were transfected using the reagent lipofectamine 3000 (Invitrogen, ThermoFisher Scientific, Rockford, IL) according to the manufacturer’s instructions. In brief, hREC (passage 6–8) were seeded at 6 × 10^5^ cells/well in six-well plates (~ 90% confluence) for the transfection. Plasmid DNA (1 µg/well) was dissolved in EBM basal medium without serum, mixed with lipofectamine 3000 (3.75 µL/well), incubated at room temperature for 15 min, and gently added to the cells. After 4 h, the medium was removed from and fresh EGM-2 medium (without VEGF added) was added to the cells. The transfection efficiency for hREC was around 15% based on immunostaining for the Myc epitope tag, and all the three DNA constructs had similar transfection efficiency. At 24 h and 48 h post transfection, the hREC were collected for analysis.

### RNA extraction, reverse transcription and real-time PCR (qRT-PCR)

Total RNA was extracted from cells using the RNAeasy mini kit (Qiagen, Valencia, CA). cDNA synthesis was performed using the iSCRIPT kit (Bio-Rad, Hercules, CA), according to the manufacturer’s instructions, using 1 µg of total RNA per reaction. For real-time PCR, reactions were performed on the LightCycler 480 II (Roche, Indianapolis, IN) using Faststart Universal SYBR Green PCR Master Mix (Roche) and 18% of cDNA per reaction. Relative gene expression was determined using the ΔΔCt method by first normalizing with the housekeeping gene HPRT1 or beta-2-microglobulin, followed by the experimental control (GFP-Ctr or untreated control). The sequences for the primers are listed in Table 1.

**Table 1:** Primers used for qRT-PCR ^39,40^

Gene name	Primer sequences (5′ to 3′)
eVEGF-38	Forward: GCACATAGGAGAGATGAGCTTC Reverse: CACCGTTACCATCGCCATTA
eVEGF-53	Forward: TGAGCTTCCTACAGCACAAC Reverse: GTTACCATCACCGTTTCCATTTC
VEGF189 (Myc tag)	Forward: GACGTGTAAATGTTCCTGCAAA Reverse: AGGAGCAACATAGTTAAGAATACCA
Human tissue factor	Forward: ACCTCGGACAGCCAACAATTCAGA Reverse: ATCCCGGAGGCTTAGGAAAGTGTT
Human E-Selectin	Forward: AGGTTCCTTCCTGCCAAGTGGTAA Reverse: ATTGAGCGTCCATAATTCAGGACA
Human VCAM-1	Forward: CTGTTGAGATCTCCCCTGGA Reverse: CGCTCAGAGGGCTGTCTATC
Human β2-Microglobulin	Forward: ACTGAATTCACCCCCACTGA Reverse: CCTCCATGATGCTGCTTACA
Human HPRT1	Forward: CCTGGCGTCGTGATTAGTGAT Reverse: AGACGTTCAGTCCTGTCCATAA
Mouse MAP1B	Forward: CCTGACTTCCACTGGTCTTTAC Reverse: GGTCTTGGGCACTCTTCTTT
Mouse Tsc1	Forward: AGCAGAGTTGAGTGAGAGAATG Reverse: GCAGCGAGAGGATGGATAAA
Mouse β2-Microglobulin	Forward: GGTCTTTCTGGTGCTTGTCT Reverse: AACTCTGCAGGCGTATGTATC
Mouse VAMP3	Forward: CTGGGAAAGAGGACTTGTGTAG Reverse: TAAGGCACACCCTGCTATTG
Mouse NRP-1	Forward: GAGGACAGAGACTGCAAGTATG Reverse: CTGAAGACACCACAGGAGAAG
Mouse GluN1	Forward: CCAGATGTCCACCAGACTAAAG Reverse: GTTCACCTTAAATCGGCCAAAG
Mouse KLF7	Forward: CCCTCTCTCCTGCATTGATTT Reverse: GTCGCTCTGTCCACTCTTAAC
Mouse VEGFR2	Forward: GCGGAGACGCTCTTCATAATA Reverse: GACAAGAAGGAGCCAGAAGAA
Mouse VEGF-A	Forward: CCAGGAGGACCTTGTGTGAT Reverse: GGGAAGGGAAGATGAGGAAG
Mouse ATF6	Forward: TGGCTTCCTTCACCACATAAA Reverse: GTATCCCTGGTTGACCCTTAAC
Mouse XBP1	Forward: CCATCACATTGCCTAGAGGATAG Reverse: AGCTGAGTGTCAAACGACAATA
Mouse DDIT3	Forward: CAGCGACAGAGCCAGAATAA Reverse: CACCGTCTCCAAGGTGAAA

### Immunoprecipitation and western blot analysis

To determine the levels of recombinant proteins associated with the cells and in the conditioned media, total cell lysate and conditioned media samples from hREC overexpressing eVEGF-38, eVEGF-53, and VEGF189 were used for immunoprecipitation. In brief, conditioned media samples were concentrated by centrifuging Amicon Ultra-0.5 Centrifugal Filter Units at 5000 × *g* at 4 °C for 30 min (3 kDa molecular weight limit, ThermoFisher). For total cell lysate, the cells were washed twice with ice-cold phosphate-buffered saline (PBS) and lysed in extraction buffer (50 mM Tris-HCl, pH 7.5, 5 mM ethylenediaminetetraacetic acid (EDTA), 100 mM NaCl, 0.5% NP40, 0.5% Triton X-100), then the lysate samples were clarified by centrifugation at 14,000 rpm at 4 °C for 10 min. The eVEGF-38, eVEGF-53, and VEGF189 proteins were immunoprecipitated from the total lysate and conditioned media samples using anti-Myc epitope antibody (Cell Signaling Technology, Danvers, MA), followed by 30 µl of protein A/G beads. These were incubated at 4 °C for 60 min with end-over-end rotation, washed three times with extraction buffer, and analyzed by sodium dodecyl sulphate polyacrylamide gel electrophoresis (SDS-PAGE) and western blotting using anti-Myc tag antibody and specific secondary antibody (Cell Signaling Technology, Danvers, MA).

For all other western blotting, medium was removed and cells were washed one time with ice-cold PBS and lysed with 200 μL of ice-cold radioimmunoprecipitation assay cell lysis buffer with protease inhibitors (Cell Signaling Technology, Danvers, MA) and NaVO3 (Sigma-Aldrich, St. Louis, MO) at 4 °C for 10 mins. The cells were then scraped and the total cell lysate transferred to 1.5 mL tubes, sonicated for 2 s and then centrifuged at 14,000 rpm at 4 °C for 15 min in order to remove cell debris. The protein concentrations of cell lysate samples were determined using the Micro BCA protein assay reagent kit (Pierce, Thermo Fisher Scientific, Rockford, IL), following the manufacturer’s instructions. Protein samples were incubated with SDS sample buffer (Bio-Rad Laboratories, Hercules, CA) for 5 min at 95 °C, then 100 µg of total protein was loaded onto a 4–20% SDS gel (Bio-Rad Laboratories, Hercules, CA) for electrophoresis and transferred to 0.22 μM nitrocellulose membranes. For detection, membranes were blocked for 1 h at room temperature with blocking buffer (5% milk in PBS), then incubated with the primary antibody in blocking solution at 4 °C overnight. The primary antibodies targeted phospho-VEGFR2 (p-VEGFR2, Y1175), VEGFR2, alpha tubulin, and Myc epitope tag (1:1000 dilution, all from Cell Signaling Technology, Danvers, MA). Membranes were washed 3 × 10 min with tris-buffered saline with Tween 20 (TBST; Cell Signaling Technology, Danvers, MA), and incubated with the secondary antibodies IRDye 800CW or IRDye 680RD (1:1000 dilution, Invitrogen) in blocking buffer for 1 h at room temperature. Membranes were washed 3 × 10 min with TBST and then scanned with a Licor Odyssey scanner (LI-COR Biosciences, Lincoln, NE). Signal intensity was determined by densitometry using ImageJ software (version 6.0, National Institutes of Health, Bethesda, MD, http://rsbweb.nih.gov/ij). Experiments were repeated at least three times using three independent lysate samples for each treatment group.

### Animals

Postnatal day (P)3 to P12 wild-type C57Bl/6 J mouse pups (Jackson Laboratory, Bar Harbor, ME) were used in this study, the animals were fed standard lab chow, received water ad libitum, and were housed in a temperature-controlled environment with a 12-hour day–night cycle. All animal procedures were reviewed and approved by the Schepens Eye Research Institute IACUC committee, and were performed in accordance with the ARVO Statement for the Use of Animals in Ophthalmic and Vision Research.

### RGC isolation and culturing

Mouse RGC were isolated using Thy1.2-conjugated magnetic beads as previously described^20^. In brief, retinas from P3, P4 or P12 mouse pups were dissected and digested with 1% papain (Worthington Biochemical, Lakewood, NJ) in Hank’s buffered saline solution containing 5 U/mL DNase for 10–30 min at 37 °C, and then dissociated cells were triturated in Papain inhibitor ovomucoid (1%; Worthington Biochemical) with 5 U/mL DNase. The suspension was centrifuged (3000 rpm, 5 min at 4 °C), and resuspended in autoMACS Rinsing Solution (Miltenyi Biotec, Auburn, CA). Cells were incubated with rat anti-Thy1.2 antibody conjugated to micrometal beads (CD90, Miltenyi Biotec, Somerville, MA) for 20 min at 4 °C. Cell suspensions were loaded onto a MS column with MACS separator (Miltenyi Biotec), and RGC were collected after washing the column for three times with autoMACS Rinsing Solution (Miltenyi Biotec).

For RGC culture, tissue culture plates were coated with poly-d-lysine (10 μg/mL; EMD Millipore, San Diego, CA) and laminin (10 μg/mL; Sigma-Aldrich, St. Louis, MO). Cells were seeded at 1 × 10^5^ cells per well in 24-well plates for staining, and 6 × 10^5^ cells per well in six-well plates for total RNA harvest for RT-qPCR analysis. Cells were cultured in Neurobasal-A medium supplemented with B27, 100 U/mL penicillin–streptomycin (Invitrogen), Glutamic acid (25 µM, Sigma-Aldrich), Glutamax (2 mM, Invitrogen), brain-derived neurotrophic factor (50 ng/ml, Peprotech, Rocky Hill, NJ), ciliary neurotrophic factor (50 ng/ml, Peprotech), forskolin (5 µM; Sigma-Aldrich), and insulin (5 µg/ml; Sigma-Aldrich) at 37 °C in a humidified tissue culture incubator with 5% CO_2_. A full medium change was performed every other day for short-term culture; for the 7-day culture, half of the medium was changed every other day.

### Production of and transduction with AAV vectors

AAV vector production and titration was performed by the Gene Transfer Vector Core at the Grousbeck Gene Therapy Center at the Schepens Eye Research Institute and Massachusetts Eye and Ear Infirmary as described previously^17,34^. In brief, all AAV preparations were made by plasmid co-transfection in HEK293 cells using polyethyleneimine. The packaging plasmid AAV Rep2-Cap2 was co-transfected with an ITR-flanked transgene (pAAV2/2.CMV.VEGF-A-38, pAAV2/2.CMV.VEGF-A-53, pAAV2/2.CMV.VEGF-A-189, pAAV2/2.CMV.GFP) and adeno-helper constructs. Three days after transfections cells and media were harvested, lysed, and digested with Benzonase. Viral vector was further purified from the lysate by tangential flow filtration and iodixanol gradient ultracentrifugation prior to reformulation in PBS^17,34^. Vectors were titrated by TaqMan PCR amplification (Applied Biosystems 7500, Life Technologies), with the primers and probes designed to detect the specific transgenes. SDS-PAGE was performed to check the purity of the vectors.

Fresh media containing the different AAV vectors (eVEGF-38/AAV2, eVEGF-53/AAV2, VEGF189/AAV2, and GFP/AAV2) at 50 multiplicity of infection were added to the RGC plated at 70% confluence in a 24-well plate. Three days later, the cells were photographed under an inverted immunofluorescence microscope for determining transduction efficiency for the GFP/AAV2 in live cells, or the cell were fixed with 4% paraformaldehyde (PFA) at room temperature for 1 h, and used for immunostaining for the three VEGF transgene constructs (anti-Myc epitope tag antibody, Cell Signaling Technology, Danvers, MA) and GFP (anti-GFP antibody, Abcam).

### Immunocytochemistry

RGC were fixed in 4% PFA at room temperature for 1 h, washed once with PBS and blocked with 2.5% normal goat serum, 2.5% normal donkey serum with 0.1% Triton-X in PBS (blocking buffer) for 1 h. Primary antibodies were rabbit anti-beta III tubulin (1:500, Abcam, Cambridge, MA), rabbit anti–synaptophysin (1:200, Abcam), rabbit anti–VEGFR2 (1:100, Abcam), rabbit anti–phospho-ERK (1:200, Cell Signaling Technology, Danvers, MA), rabbit anti–phospho-Akt (1:200, Cell Signaling Technology), rabbit anti–active caspase-3 (1:200, Cell Signaling Technology), mouse anti-Myc epitope tag (1:500, Cell Signaling Technology), mouse anti-GAP-43 (1:200, Sigma-Aldrich), and mouse anti-MAP1B (1:50, Santa Cruz, CA), and the cells were incubated with the primary antibodies at 4 °C overnight. Secondary antibodies were goat anti-mouse or donkey anti-rabbit conjugated to Alexa Fluor 488, − 594, or − 647 (1:500, Invitrogen). After three PBS washes, the RGC were mounted in medium containing 50% glycerol, 50% PBS, and 0.04% sodium azide and viewed on a Axioskop 2 Mot Plus microscope equipped with an Axiocam MRm monochrome color camera (Carl Zeiss Inc.). The density of the synaptophysin-positive cluster was determined by counting the number of synaptophysin puncta per 10 μm neurite from individual RGC using Image-Pro Plus 6.0 software (Media Cybernetics, Rockville, MD) according to published methods^35,36^.

### Eyecup differentiation and RGC analysis by flow cytometry

Retinal organoids were differentiated from mouse embryonic stem cells (mES) from the C57Bl6 background as described^37^. In brief, mES cells were dissociated and the suspension was placed in V-shaped wells in polypropylene flasks for embryoid body formation in optic vesicle medium (2000 cells in 50 µL). The following day, neural differentiation was induced by supplementing the medium with 1% matrigel (Corning, Tewksbury, MA). Only the aggregates forming optic vesicles and optic cups were selected for the experiment.

Starting on day 9 of differentiation, the aggregates were transferred to non-adherent Petri dishes and fed with Optic Cup (OC) medium containing NS21 supplement as described^37^. At day 21 of differentiation, the organoids were collected for AAV transduction.

For transduction 12 retinal organoids were pooled together and washed once in Hank's Balanced Salt Solution, combined with the AAV viruses (GFP/AAV2, eVEGF-53/AAV2, or VEGF189/AAV2, 4 × 10^10^ GC per condition), and incubated for 20 min on an orbital shaker at room temperature, then transferred to the incubator. Six hours later OC medium was added and organoids were cultured for another 7 days with the medium changed on every other day. At the terminal points the retinal organoids were collected and dissociated with Trypsin-EDTA, with the resulting cell suspension used for the flow cytometry as described previously^38^.

Analysis of transduction efficiency and retinal ganglion cell counts used the primary antibodies chicken anti-GFP (1:2000, Abcam), mouse anti-Myc epitope tag (1:300, Cell Signaling), rabbit anti-RBPMS (1:400, Chemicon), and rabbit anti-Brn3A (1:400, Chemicon), and the corresponding secondary antibodies Alexa488 anti-mouse, Alexa488 anti-chicken, Alexa647 anti-rabbit (all 1:500, Jackson Immuno). For immunohistological analysis of transgene expression, retinal organoids were fixed in 4% PFA and processed for whole-mount staining using anti-Myc tag (Cell Signaling), anti-GFP (Abcam), anti-RBPMS (Chemicon), and anti-Brn3A (Chemicon) antibodies with procedures as described above. The percentage of viable RGC in the retinal organoids was determined by flow cytometry (RBPMS−positive cells)/(total cells).

### Viability assays

To determine the percentage of viable RGC in culture, 3 days after AAV transduction cell viability was determined by dividing the number of beta III tubulin-positive & DAPI-positive cells by the total number of DAPI-positive cells, and by dividing the number of beta III tubulin-positive & transgene-positive & DAPI-positive cells by the total number of DAPI-positive cells. For VEGF121 protein treatment (105 ng/mL, Cell Signaling Technology), RGC were treated at DIV 2 for 24 h. Cells bearing neurites were photographed using an Axioskop 2 Mot Plus microscope (Carl Zeiss, Inc., Throwood, NY) equipped with an Axiocam MRm monochrome color camera (Carl Zeiss Inc.). Axiovision 4.9.1 (Carl Zeiss Inc.) was used for image acquisition.

To determine RGC viability upon exposure to cytotoxic stimuli, RGC were treated 3 days after AAV transduction with H_2_O_2_ (Sigma-Aldrich) at 1 μM, 10 μM, or 100 μM final concentration for 5 h, or with NMDA (Cayman Chemical) at 50 μM, 100 μM, or 500 μM final concentration for 12 h. To determine the role of the PI3K/Akt pathway in RGC survival, the PI3K small-molecule inhibitor LY294002 (10 μM, Cell Signaling Technology) was added at the same time as the H_2_O_2_. To determine the roles of VEGFR2 activation and PI3K/Akt signaling in neurite outgrowth, RGC were treated with the VEGFR2-selective small-molecule receptor tyrosine kinase inhibitor sunitinib malate (1 µM, Selleck Chemicals, Houston, TX) or the PI3K small-molecule inhibitor LY294002 (1 µM, Cell Signaling Technology) at DIV 2 for 24 h.

Apoptotic cells were detected using the In Situ Cell Death Detection Kit (TUNEL, Roche) according to manufacturer’s instructions. RGC were fixed in 4% PFA for 10 min and permeabilized with 0.1% Triton X-100 and 0.1% sodium citrate in PBS before proceeding with the TUNEL reaction. Cells positive for both DAPI and TUNEL were counted as apoptotic, and the number of viable cells per field was determined by subtracting the number of TUNEL-positive cells from the number of DAPI-positive cells. The percentage of viable RGC was determined by dividing the number of TUNEL-negative & transgene-positive & DAPI-positive cells by the total number of DAPI-positive cells. Apoptotic cells were also detected by immunostaining of cleaved active caspase-3 (1:200, Cell Signaling Technology), and the percentage of viable RGC was determined by dividing the number of cleaved caspase-3-negative & transgene-positive & DAPI-positive cells by the total number of DAPI-positive cells.

### Measurement of [Ca^2+^]i

Three days after AAV transduction, RGC were incubated for 1 h at 37 °C with Krebs–Ringer bicarbonate buffer with HEPES (KRB-HEPES), with and without 1.0 mM Ca^2+^, plus 0.5% bovine serum albumin containing 0.5 µM fura 2/AM (Invitrogen), 8 µM pluronic acid F127, and 250 µM sulfinpyrazone, followed by washing in KRB-HEPES containing sulfinpyrazone. Calcium measurements were made with a ratio imaging system (In Cyt Im2, Intracellular Imaging, Cincinnati, OH) using excitation wavelengths of 340 nm and 380 nm and an emission wavelength of 505 nm. Following addition of the NMDA receptor agonist NMDA to the RGC, calcium influx ([Ca^2+^]i) was measured in real-time. At least five cells were measured from each group per field per experiment, and at least three independent wells were used for each group.

### SEM

To create coverslips with grids for SEM, nickel 200-mesh alpha-numeric finder grids (G200F1-Ni, Electron Microscopy Sciences, Hatfield, PA) were collected on thin formvar film mounted on 12 mm diameter coverslips. The coverslips were then carbon-coated using a Gatan 681 high resolution ion beam coater (Gatan, Inc., Pleasanton, California) and sterilized using 70% ethyl alcohol in distilled water prior to use for RGC culture. RGC were seeded at 100,000 cells per coverslip; both coverslips with grids and regular coverslips without grids were used.

Three days after AAV transduction, RGC were fixed in 4% PFA and processed for immunocytochemistry using anti-Myc antibody as described above. Fluorescence imaging was conducted using an EVOS FL Auto microscope (Thermo Fisher Scientific). After fluorescence imaging the samples were treated with half-strength Karnovsky’s fixative (2% PFA + 2.5% glutaraldehyde in 0.1 M sodium cacodylate buffer, pH 7.4) for 3 h at room temperature, rinsed with 0.1 M sodium cacodylate buffer and post-fixed with 1% osmium tetroxide in 0.1 M sodium cacodylate buffer for 1 h. The RGC samples were dehydrated with graded ethyl alcohol solutions, placed in several changes of 2,2-dimethoxypropane and then air dried, mounted onto an aluminum stub and chromium-coated using a Gatan 681 high resolution ion beam coater (Gatan, Inc., Pleasanton, CA). SEM images were acquired using a JEOL JSM-7401F field emission scanning electron microscope (JEOL USA, Inc., Peabody, MA) at 5 kV. For samples on coverslips with and without grids, correlative SEM images were acquired at the exact locations at which transgene-expressing RGC were identified by immunostaining.

### Statistical analysis

Statistical analyses were performed using GraphPad Prism software version 4 (GraphPad Software, La Jolla, CA). Unpaired two-tailed *t* test or one-way analysis of variance followed by a Dunnett’s post hoc test were used to compare the different test groups with the control as indicated. The sample size (*n*) for each experiment is noted in the figure legends. For all comparisons, values of *P* < 0.05 were considered statistically significant. Data are shown as mean ± SEM unless otherwise noted.

## Electronic supplementary material

Supplemental Figures S1-5

Supplementary figure legends

## References

[1] Mackenzie F, Ruhrberg C (2012). Diverse roles for VEGF-A in the nervous system. Development.

[2] Soker S, Takashima S, Miao HQ, Neufeld G, Klagsbrun M (1998). Neuropilin-1 is expressed by endothelial and tumor cells as an isoform-specific receptor for vascular endothelial growth factor. Cell.

[3] Kim I (1999). Constitutive expression of VEGF, VEGFR-1, and VEGFR-2 in normal eyes. Invest. Ophthalmol. Vis. Sci..

[4] Ogunshola OO (2002). Paracrine and autocrine functions of neuronal vascular endothelial growth factor (VEGF) in the central nervous system. J. Biol. Chem..

[5] Foxton RH (2013). VEGF-A is necessary and sufficient for retinal neuroprotection in models of experimental glaucoma. Am. J. Pathol..

[6] Ferrara N (2010). Vascular endothelial growth factor and age-related macular degeneration: from basic science to therapy. Nat. Med..

[7] Nishijima K (2007). Vascular endothelial growth factor-A is a survival factor for retinal neurons and a critical neuroprotectant during the adaptive response to ischemic injury. Am. J. Pathol..

[8] Foxton R, Osborne A, Martin KR, Ng YS, Shima DT (2016). Distal retinal ganglion cell axon transport loss and activation of p38 MAPK stress pathway following VEGF-A antagonism. Cell Death Dis..

[9] Saint-Geniez M (2008). Endogenous VEGF is required for visual function: evidence for a survival role on muller cells and photoreceptors. PLoS. ONE.

[10] Ferrara N, Gerber HP, LeCouter J (2003). The biology of VEGF and its receptors. Nat. Med..

[11] Ishida S (2003). VEGF164-mediated inflammation is required for pathological, but not physiological, ischemia-induced retinal neovascularization. J. Exp. Med..

[12] Choi C, Nitabach MN (2013). Membrane-tethered ligands: tools for cell-autonomous pharmacological manipulation of biological circuits. Physiology.

[13] Ruch C, Skiniotis G, Steinmetz MO, Walz T, Ballmer-Hofer K (2007). Structure of a VEGF-VEGF receptor complex determined by electron microscopy. Nat. Struct. Mol. Biol..

[14] Ng YS, Rohan R, Sunday ME, Demello DE, D’Amore PA (2001). Differential expression of VEGF isoforms in mouse during development and in the adult. Dev. Dyn..

[15] Park JE, Keller GA, Ferrara N (1993). The vascular endothelial growth factor (VEGF) isoforms: differential deposition into the subepithelial extracellular matrix and bioactivity of extracellular matrix-bound VEGF. Mol. Biol. Cell.

[16] Wassmer SJ, Carvalho LS, Gyorgy B, Vandenberghe LH, Maguire CA (2017). Exosome-associated AAV2 vector mediates robust gene delivery into the murine retina upon intravitreal injection. Sci. Rep..

[17] Lock M (2010). Rapid, simple, and versatile manufacturing of recombinant adeno-associated viral vectors at scale. Hum. Gene. Ther..

[18] Guo C (2018). IGFBPL1 regulates axon growth through IGF-1-mediated signaling cascades. Sci. Rep..

[19] Leaver SG (2006). AAV-mediated expression of CNTF promotes long-term survival and regeneration of adult rat retinal ganglion cells. Gene Ther..

[20] Jiao J (2005). Bcl-2 enhances Ca(2 + ) signaling to support the intrinsic regenerative capacity of CNS axons. EMBO J..

[21] Zhang SX, Ma JH, Bhatta M, Fliesler SJ, Wang JJ (2015). The unfolded protein response in retinal vascular diseases: implications and therapeutic potential beyond protein folding. Prog. Retin. Eye. Res..

[22] Jin KL, Mao XO, Greenberg DA (2000). Vascular endothelial growth factor: direct neuroprotective effect in in vitro ischemia. Proc. Natl. Acad. Sci. USA.

[23] Thomas CN, Berry M, Logan A, Blanch RJ, Ahmed Z (2017). Caspases in retinal ganglion cell death and axon regeneration. Cell. Death Discov..

[24] Simons M, Gordon E, Claesson-Welsh L (2016). Mechanisms and regulation of endothelial VEGF receptor signalling. Nat. Rev. Mol. Cell Biol..

[25] Frank L, Ventimiglia R, Anderson K, Lindsay R, Rudge J (1996). BDNF down-regulates neurotrophin responsiveness, TrkB protein and TrkB mRNA levels in cultured rat hippocampal neurons. Eur. J. Neurosci..

[26] Dekeyster E (2015). Tackling glaucoma from within the brain: an unfortunate interplay of BDNF and TrkB. PLoS ONE.

[27] Sondell M, Lundborg G, Kanje M (1999). Vascular endothelial growth factor has neurotrophic activity and stimulates axonal outgrowth, enhancing cell survival and Schwann cell proliferation in the peripheral nervous system. J. Neurosci..

[28] Storkebaum E, Carmeliet P (2004). VEGF: a critical player in neurodegeneration. J. Clin. Invest..

[29] Brockington A (2010). Downregulation of genes with a function in axon outgrowth and synapse formation in motor neurones of the VEGFdelta/delta mouse model of amyotrophic lateral sclerosis. BMC Genomics.

[30] Sun Y (2003). VEGF-induced neuroprotection, neurogenesis, and angiogenesis after focal cerebral ischemia. J. Clin. Invest..

[31] Laub F (2005). Transcription factor KLF7 is important for neuronal morphogenesis in selected regions of the nervous system. Mol. Cell Biol..

[32] Moore DL (2009). KLF family members regulate intrinsic axon regeneration ability. Science.

[33] Veldman MB, Bemben MA, Thompson RC, Goldman D (2007). Gene expression analysis of zebrafish retinal ganglion cells during optic nerve regeneration identifies KLF6a and KLF7a as important regulators of axon regeneration. Dev. Biol..

[34] Zinn E (2015). In silico reconstruction of the viral evolutionary lineage yields a potent gene therapy vector. Cell Rep..

[35] Roque PJ, Guizzetti M, Costa LG (2014). Synaptic structure quantification in cultured neurons. Curr. Protoc. Toxicol..

[36] Wu KW (2017). Neurovascular interaction promotes the morphological and functional maturation of cortical neurons. Front. Cell Neurosci.

[37] Eiraku M (2011). Self-organizing optic-cup morphogenesis in three-dimensional culture. Nature.

[38] Baranov P, Regatieri C, Melo G, Clissold H, Young M (2013). Synthetic peptide-acrylate surface for self-renewal of human retinal progenitor cells. Tissue Eng. Part. C. Methods.

[39] Nugent HM (2012). Ultrasound-guided percutaneous delivery of tissue-engineered endothelial cells to the adventitia of stented arteries controls the response to vascular injury in a porcine model. J. Vasc. Surg..

[40] Park-Windhol C (2017). Endomucin inhibits VEGF-induced endothelial cell migration, growth, and morphogenesis by modulating VEGFR2 signaling. Sci. Rep..

