# Supplementary figures and images for: Novel engineered, membrane-localized variants of vascular endothelial growth factor (VEGF) protect retinal ganglion cells: a proof-of-concept study
Source: Cell Death Dis. 2018 Oct 3;9(10):1018. doi: 10.1038/s41419-018-1049-0 (PMC6170416; doi:10.1038/s41419-018-1049-0)

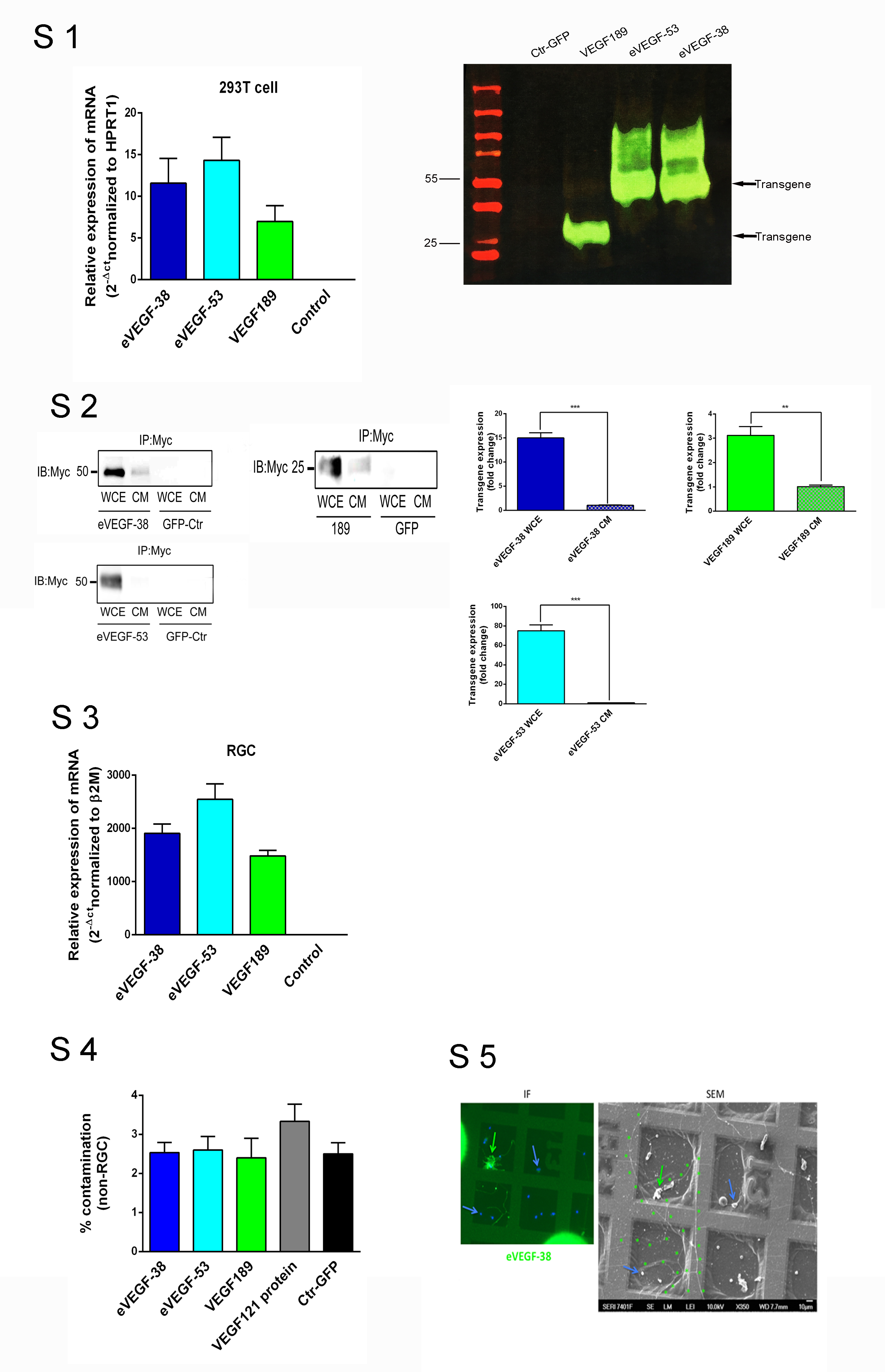

Supplement: Supplementary file 1 — Supplemental Figures S1-5 [file 41419_2018_1049_MOESM1_ESM.tif]
