# Supplementary material for: Novel engineered, membrane-localized variants of vascular endothelial growth factor (VEGF) protect retinal ganglion cells: a proof-of-concept study
Source: Cell Death Dis. 2018 Oct 3;9(10):1018. doi: 10.1038/s41419-018-1049-0 (PMC6170416; doi:10.1038/s41419-018-1049-0)
Supplement: Supplementary file 2 — Supplementary figure legends [file 41419_2018_1049_MOESM2_ESM.docx]

**Supplemental Figure S1**: **VEGF transgenes delivered by plasmid transfection are expressed by Hek-293T cells**. Left panel, Hek-293T cells were transfected with eVEGF-38, eVEGF-53, VEGF189 or GFP plasmids, then the expression levels for the transgenes mRNA were determined by quantitative real-time qRT-PCR at 24 hours post transfection, using specific primer sets that can amplify the three different VEGF constructs individually. Right panel, western blot showing expression of the eVEGF-38, eVEGF-53 and VEGF189 proteins at 48 hours using antibodies to the Myc tag. Data = means ± SEM. n = three independent experiments.

**Supplemental Figure S2**: **The eVEGF constructs are mostly cell-associated proteins.** Left, immunoprecipitation (IP) and western blot analysis showing the expression of eVEGF-38, eVEGF-53 and VEGF189 proteins in the total cell lysates, while only a small amount of each protein was detected in the conditioned medium. Right, quantification of the western blot data. WCE, whole cell extract; CM, conditioned media; IB, immune blotting. ***P < 0.001, **P < 0.01, unpaired 2-tail t-test, n = three independent experiments. Data = means ± SEM.

**Supplemental Figure S3**: **VEGF transgenes delivered by AAV2 transduction are expressed by primary mouse RGC.** Primary mouse RGC isolated from P3 pups were transduced with eVEGF-38, eVEGF-53, VEGF189 or GFP, and the expression levels for VEGF mRNA were determined by quantitative real-time qRT-PCR at three days post transduction. n = two independent experiments. Data = means ± range.

**Supplemental Figure S4: The primary culture of mouse RGC contains at least 96% RGC.** The purity of the primary mouse RGC isolated from P3 pups in culture three days post transduction with eVEGF-38, eVEGF-53, VEGF189, GFP or VEGF121 protein treatment was assessed by immunostaining (β-III tubulin). The percentage of non-RGC cells in each group was below 4%. n = three independent experiments. Data = means ± SEM.

**Supplemental Figure S5**: **Correlative imunofluorescent (IF) and scanning electronic microscopy (SEM) for P3 RGC expressing eVEGF-38.** Primary RGC were plated on a cover slip with grid, transduced with eVEGF-38/AAV2 and, on day three, harvested for IF and imaging followed by SEM. RGC expressing eVEGF-38 (green arrow) produced long axons (green dots) whereas RGC not expressing eVEGF-38 (blue arrows) produced only short neurites. SEM at 400x magnification.
